# Validity of somatic cell count as indicator of pathogen-specific intramammary infections

**DOI:** 10.4102/jsava.v88i0.1465

**Published:** 2017-04-13

**Authors:** Inge-Marié Petzer, Joanne Karzis, Edward F. Donkin, Edward C. Webb, Eric M.C. Etter

**Affiliations:** 1 Department of Production Animal Studies, University of Pretoria, South Africa; 2 Department of Animal and Wildlife Sciences, University of Pretoria, South Africa; 3 Department Environment and Societies, French Agricultural Research Centre for International Development (CIRAD), France

## Abstract

The objective of this study was to determine whether somatic cell count (SCC) was an effective test, with a sensitivity exceeding 85%, to determine species-specific bacterial infections. In addition, the relation between the SCC and various udder pathogen groups was investigated. SCC thresholds of greater than 200 000 cells/mL were used in quarter and greater than 150 000 cells/mL in composite milk samples. A retrospective study was conducted on a data set for 89 635 quarter and 345 467 composite cow milk samples. Eleven SCC threshold values were used to evaluate the diagnostic efficacy for the following bacteria: Gram-positive major pathogens: *Staphylococcus aureus, Streptococcus agalactiae, Streptococcus dysgalactiae* and *Streptococcus uberis*; Gram-negative major pathogens: *Escherichia coli, Klebsiella pneumonia* and *Serratia* spp.; minor pathogens: coagulase-negative staphylococci, *Micrococcus* spp., *Staphylococcus pseudintermedius, Streptococcus pyogenes, Enterococcus faecalis, Enterococcus canis, Trueperella pyogenes* and other *Enterobacteriaceae.* Sensitivity and specificity were calculated taking the effect of clustering into account with quarter milk samples. Most samples yielding major Gram-positive pathogens (88.9% in quarter and 79.9% in composite samples) and minor pathogens (61.4% in quarter and 51.7% in composite samples) had SCC greater than 200 000 cells/mL. Sensitivity of the SCC test to detect major pathogens at an SCC threshold of greater than 200 000 cells/mL in quarter samples and greater than 150 000 cells/mL in composite milk samples was 88.2% and 84.2%, respectively, but specificity was low (57.7% and 52.8%, respectively).

## Introduction

Mastitis remains the most costly disease in dairy cattle (Geary et al. 2012). Subclinical mastitis is considered the most important form of mastitis, as it has been estimated to be responsible for more than 80% of economic mastitis losses (Giesecke, Du Preez & Petzer 1994; Shim, Shanks & Morin 2004) due to long-term reduction in milk quality and production (Halasa et al. 2009; Holland et al. 2015). Somatic cell counts (SCC) have generally been accepted and used as a measure of inflammation (Barkema et al. 1999; Biggs 2009; Heeschen 2010), as a measure of the severity of intramammary infection (IMI) (Harmon 1994) and as an indicator of economic losses (DeGraves & Fetrow 1993). The SCC threshold value used to describe normal milk, IMI and subclinical mastitis has, however, often been and still is controversial (Heeschen 2010). Dohoo et al. (2011) included the following criteria for diagnosing IMI: the number of colonies isolated, whether the organism was recovered in pure culture, and whether or not IMI was present with an increased SCC. Their conclusions were based on a study in which culture results of single milk samples were compared with a set of triplicate quarter milk samples (used as ‘gold standard’). The authors concluded that results of a single sample may be used for the diagnosis of IMI in quarter milk when a minimum of one or two colonies were recorded without taking the SCC into consideration. The term ‘IMI’ used in this study refers to the presence of a bacterial species isolated from a milk sample as a pure culture, regardless of the level of SCC. A pure culture was defined when only one species grew on an inoculum.

Early detection of subclinical mastitis remains important for treatment success (Ruegg 2004), particularly in cases of contagious pathogens (*Staphylococcus aureus* and *Streptococcus agalactiae*) where the infected udder is the primary source of infection for other cows (Ruegg 2004; Fox & Gay 1993; Keefe 1997; Neave et al. 1969). However, performing microbiology on milk samples of all cows in a herd can be expensive, and therefore, other effective diagnostic and screening tests are essential to identify affected cows for further investigation. The principal reason for increased SCCs in milk of dairy cows remains IMI (Giesecke et al. 1994). In 2001, the National Mastitis Council (USA) stated that when a quarter SCC is equal to or exceeds 200 000 cells/mL, the likelihood is high that it is infected or recovering from an infection (National Mastitis Council 2001). A study conducted on a large data set that did not differentiate between udder pathogens and where results were obtained using Youden’s index and ROC graphs indicated that 200 000 cells/mL or more in quarter milk samples and 150 000 cells/mL or more in composite milk samples were the optimum SCC to indicate IMI and culturing of positive samples (Petzer et al. 2017).

Although more than 100 different microorganisms can cause mastitis, only a few species of staphylococci, streptococci and Gram-negative organisms are currently considered to be of economic importance (Zadoks & Fitzpatrick 2009). There have been changes in the relative proportion of mastitis pathogens over the past 60 years in First World countries due to changes in herd and parlour management, udder health monitoring, treatments and differences in genetics (Zadoks & Fitzpatrick 2009). Research in countries practising good udder health has recorded a lower incidence of mastitis caused by contagious bacteria but increases in Gram-negative bacteria. In South Africa, most cases of subclinical mastitis have been caused by coagulase-negative staphylococci (CNS), *S. aureus, S. agalactiae* and *Streptococcus uberis* (Petzer et al. 2009). CNS, regarded as minor pathogens, are now causing an increasing number of clinical mastitis cases and increasingly high SCC (Petzer et al. 2009; Zadoks & Watt 2009).

The primary objective of this study was to determine whether or not the SCC test could be used with a reasonable level of accuracy (sensitivity > 85%) to identify species-specific IMI. The study investigated how SCC levels differ between the various udder pathogens and pathogen groups in both quarter milk samples and composite milk samples. In addition, the percentage of infection with a species with SCC below 200 000 cells/mL in quarter milk samples and below 150 000 cells/mL in composite milk samples was quantified.

Decisions in the field need to rely on current farm information to predict future events accurately so that measures can be taken to prevent disease in advance. Early identification and dealing with cows positive for contagious udder pathogens can lower the risk of transmission for new infections significantly (Petzer et al. 2017). An effective, inexpensive test is therefore required to aid in early detection of the presence of bacteria.

## Methods

A retrospective study was conducted of milk cultures of lactating cows obtained from South African commercial dairy herds over a period of 4 years. Samples originated from approximately 830 South African dairy herds that submitted samples as part of their routine udder health monitoring programme and also from herds with increased bulk milk somatic cell count (BMSCC) who had sought help. The South African national average daily milk yield was 20.2 kg (Lactodata 2013) and lactating cow numbers varied from approximately 30–1700 cows in herds tested in this data set. Intervals for herd examinations ranged from monthly to annually and bi-annually, or in some cases only as a once-off test. The study population included different dairy breeds and cows differed in parity, milk yield and days in milk.

Quarter and composite milk samples were investigated. Milk samples were taken by professional samplers or milkers trained according to a standard operating procedure (Giesecke et al. 1994). Prior to sampling, first milk was stripped from all quarters and teat ends were carefully cleaned and disinfected with methylated alcohol. Approximately 10 mL of foremilk was collected aseptically into sterile marked sample tubes and kept refrigerated until shipment. In the case of composite milk samples, the same procedure was followed, only approximately equal volumes of milk from each of the four quarters were collected in one sample tube. Samples were transported on ice to reach the Milk Laboratory at the University of Pretoria (Faculty of Veterinary Science) within 48 h after sampling. Temperatures and conditions such as sample tube cleanliness and appearance were noted on arrival at the laboratory, and samples that were spoiled or of doubtful quality were not processed. Samples were plated out at the laboratory on the day of their arrival.

A total of 95 228 quarter milk samples and 386 031 composite milk samples obtained from routine herd investigations were initially under consideration for use, of which 5.9% and 10.5%, respectively, were defined as being unsuitable and were excluded from the data set. Samples were regarded as unsuitable for this study when any visible abnormalities such as dirt, flakes or blood were detected, when cultures indicated contamination or mixed bacterial growth (≥ 2 bacterial species), or when data were incomplete. In cases where two major pathogens were isolated from the same sample, an identifying code was used in order to be able to use this information for herd management decisions, but this information was not included in the data set. The final data set comprised 89 638 quarter milk samples and 345 461 composite milk samples.

Milk from samples was plated out on bovine blood tryptose agar plates (Oxoid, supplied by Quantum Biotechnologies [Pty] Ltd, Ferndale, South Africa). Inoculated agar plates were incubated aerobically at 37 °C ± 1 °C and examined after 18–24 and 48 h. All samples in the current data set were diagnosed by using one or more colonies in cases where *S. aureus* was suspected and two or more in all other cases. Only pure cultures were used. Colonies were initially identified based on colony morphology, haemolysis and potassium hydroxide (KOH) test results (International Dairy Federation 1981; Sears et al. 1993). The catalase reaction was used to differentiate between Gram-positive staphylococci and streptococci. Staphylase, a coagulase test (Oxoid, supplied by Quantum Biotechnologies [Pty] Ltd, Ferndale, South Africa), was used to distinguish between coagulase-positive and coagulase-negative staphylococci. Maltose agar plates (Merck NT Laboratory Supplies, Halfway House, South Africa) and Staph API (Biomerieux South Africa [Pty] Ltd, Randburg, South Africa) were used for further identification of staphylococci. Catalase-negative streptococci were differentiated into the various Lancefield groups using a Strepkit (Oxoid, supplied by Quantum Biotechnologies [Pty] Ltd, Ferndale, South Africa). Gram-negative organisms were diagnosed using DNase and MacConkey agar (Oxoid supplied by Quantum Biotechnologies [Pty] Ltd, Ferndale, South Africa) and API 20E (Biomerieux South Africa [Pty] Ltd, Randburg, South Africa). *Staphylococcus aureus* isolates were further identified by phage typing (Blair & Williams 1961). Typing was performed by using an international set of 23 phages. The strains were typed as one of four groups or as non-allocated. All *S. aureus* isolates that were identified as being from phage group 3 were indicated by the abbreviation STH whereas all those that were not from this group were identified as STA. Phage typing was performed to differentiate between the *S. aureus* isolates for another study investigating the possible role of reverse zoonosis in udder health.

Somatic cells were counted by fluoro-opto-electronics using a Fossomatic 5000 (Rhine Ruhr). SCC was evaluated by comparing the presence or absence of 8 and 10 specific pathogens isolated at 11 threshold levels in quarter and composite sample types, respectively. SCC thresholds were used with increments of 50 000 cells/mL – 500 000 cells/mL; thereafter, the two thresholds were 750 000 cells/mL and above.

IMI was diagnosed in single quarter milk samples at any level of SCC and only using pure cultures when at least one or more *S. aureus* colonies and two or more colonies of any other bacteria were isolated (Dohoo et al. 2011). In the case of composite cow milk samples, the same principle was used but referred to as culture-positive samples. Two by two tables were generated using the GenStat programme (Payne et al. 2012) and the positive likelihood ratio was used.

In the case of composite milk samples sensitivity, specificity (95% confidence intervals [CI]) of binomial proportions around these estimates was determined using the Mid-P exact test from the OpenEpi freeware programme. Sample dependency of SCC values within each cow was modelled to take into account individual cow factors that may affect quarter samples. An estimator of cluster variance was determined and the 95% CI were adjusted for clustering by using the design effect (cluster variance or sample random sample variance; Scheaffer, Mendenhall & Ott 1996).

## Results

### Quarter milk samples

Bacteria were isolated from 33.9% of quarter milk samples examined. Of these culture-positive samples, 12.43% were major Gram-positive, 0.3% major Gram-negative and 21.2% minor pathogens. The most frequently isolated bacteria were CNS (19.2%), *S. aureus* (STA and STH) (6.8%), *S. agalactiae* (2.1%), *S. uberis* (2.5%) and *Streptococcus dysgalactiae* (1.1%). The percentage of quarter milk samples with an SCC in excess of 200 000 cells/mL was 48.8% and bacteria were isolated from 49.9% of these samples (Table 1).

**TABLE 1:** Bacteriologically negative and positive quarter milk samples indicating pathogen-specific bacteria and groups isolated from samples with and without somatic cell counts above 200 000 cells/mL milk.

Bacteria isolated	Total numbers of samples from which bacteria were isolated	Bacterial species as a % of the total samples	Bacterial species as a % of bacteria isolated	Number of quarters with an SCC > 200 000 cells/mL per bacterial species	As a % of all quarters with an SCC > 200 000 cells/mL per bacterial species†	As a % of bacteriological diagnoses with SCC > 200 000 cells/mL
*Staphylococcus aureus* (STA)	3657	4.1	12.0	3125	7.2	85.5
*Staphylococcus aureus* (STH)	2430	2.7	8.0	2286	5.2	94.1
*Streptococcus agalactiae*	1875	2.1	6.2	1707	3.9	91.0
*Streptococcus uberis*	2212	2.5	7.3	1856	4.3	83.9
*Streptococcus dysgalactiae*	964	1.1	3.2	924	2.1	95.9
**Major Gram-positive pathogens**	**11 138**	**12.4**	**36.6**	**9898**	**22.7**	**88.9**
*Escherichia coli*	141	0.2	0.5	128	0.3	90.8
*Klebsiella pneumonia*	45	0.1	0.2	42	0.1	93.3
*Serratia* spp.	102	0.1	0.3	99	0.2	97.1
**Major Gram-negative pathogens**	**288**	**0.3**	**1.0**	**269**	**0.6**	**93.4**
**All major pathogens**	**11 426**	**12.8**	**37.6**	**10 167**	**23.3**	**89.0**
*Staphylococcus pseudintermedius*	445	0.5	1.5	237	0.5	53.3
*Coagulase-negative staphylococci*	17 249	19.2	56.7	10 352	23.7	60.0
*Streptococcus pyogenes*	56	0.1	0.2	53	0.1	94.6
*Enterococcus faecalis*	651	0.7	2.1	557	1.3	85.6
*Enterococcus canis*	33	0.0	0.1	26	0.1	78.8
*Trueperella pyogenes*	19	0.0	0.1	16	0.0	84.2
*Micrococcus* spp.	141	0.2	0.5	104	0.2	73.8
*Other Gram-negative bacteria*	379	0.4	1.3	309	0.7	81.5
**All minor pathogens**	**18 973**	**21.2**	**62.4**	**11 654**	**26.7**	**61.4**
All culture-negative samples	59 236	66.1	-	21 876	50.1	36.9
**All culture-positive samples**	**30 399**	**33.9**	-	**21 821**	**49.9**	**71.8**
**All samples tested**	**89 635**	-	-	**43 697**	-	**48.8**

*Source*: Milk laboratory, Faculty of Veterinary Science, University of Pretoria

† , The number of quarter milk samples, per pathogen group or species, that had a somatic cell counts of below 200 000 cells/mL expressed as a percentage of the total quarters (43 697) identified with somatic cell counts below 200 000 cells/mL.

SCC, somatic cell counts.

The cumulative percentages of the eight most prominent bacterial species isolated in quarter milk samples are indicated at 11 different SCC threshold levels. Less than 10% of the following bacterial species were isolated at an SCC of below 200 000 cells/mL: *S. dysgalactiae* (4.2%), *S. aureus* (STH) (5.9%) and *S. agalactiae* (9.0%). In contrast, 40.0% of CNS and 46.2% *Staphylococcus pseudintermedius* were isolated at an SCC of below 200 000 cells/mL (Table 2).

**TABLE 2:** Cumulative percentages of eight mastitis pathogens and culture-negative samples isolated from quarter samples distributed on various cell count thresholds.

SCC × 10^3^ cells/mL	% CNS	% STA	% STH	% STI	% SAG	% SUB	% SDY	% SFA	% no growth
50	14.8	7.1	3.0	22.4	2.7	5.2	1.4	4.0	34.6
100	26.2	11.7	4.8	34.0	5.8	10.0	2.8	7.9	40.3
150	35.0	12.7	5.5	42.2	7.1	14.2	3.6	11.4	56.9
200	40.0	14.6	5.9	46.7	9.0	16.1	4.2	14.4	63.1
250	47.3	21.1	10.4	53.9	12.5	21.1	6. 7	20.3	69.0
300	52.1	23.1	13.1	59.2	15.5	24.3	8.7	24.3	72.2
350	55.9	24.7	14.5	63.2	18.7	27.8	10.6	28.1	74.6
400	59.2	26.5	15. 5	66.4	21.9	30.9	12.7	31.6	76.6
450	62.1	28.5	17.4	69.2	25.2	33.2	14.0	34.5	78.3
500	64.6	30.1	18.8	73.2	26.8	35.8	16.0	36.6	79.7
750	73.3	36.2	25.8	79.3	37.5	46.4	24.6	45.3	84.7
Sample size	17 249	3657	2430	445	1875	2212	964	651	59236

*Source*: Milk laboratory, Faculty of Veterinary Science, University of Pretoria

*n* = 89 635.

SCC, somatic cell counts; CNS, coagulase-negative staphylococci; STA and STH, *Staphylococcus aureus*; STI, *Staphylococcus pseudintermedius*; SAG, *Streptococcus agalactiae*; SUB, *Streptococcus uberis*; SDY, *Streptococcus dysgalactiae*; SFA, *Enterococcus faecalis*; no growth, culture-negative samples.

Sensitivity (95% CI) at an SCC threshold of 200 000 cells/mL for all bacteria that were isolated (Table 3) and for major and minor pathogen groups identified was 70.8%, 88.2% and 60.7%, and specificity was 66.6%, 57.7% and 55.6%, respectively. The sensitivity for detecting *S. dysgalactiae* (95.6%), *S. aureus* (STH) (93.3%), *S. agalactiae* (90.5%) and the major Gram-negative pathogen group (93.1%) using SCC was high. The highest sensitivity obtained with the SCC test indicating a minor pathogen species was 85.4% for detecting *Enterococcus faecalis.* Positive likelihood ratios at 200 000 cells/mL to indicate major and minor pathogens were 2.085 and 1.369, respectively (Table 3).

**TABLE 3:** Sensitivity and specificity with 95% confidence intervals for pathogen-specific detection using somatic cell counts threshold of 200 000 cells/mL milk in quarter milk samples and positive likelihood ratios at somatic cell counts thresholds of 200 000 cells/mL.

Number of samples	Bacteria isolated	Sensitivity (95% LCI and UCI)^a^	Specificity (95% LCI and UCI)^a^	Positive likelihood ratio
89 635	All bacteria isolated	0.71 (0.70, 0.72)	0.67 (0.64, 0.69)	2.12
3657	*Staphylococcus aureus* (STA)	0.85 (0.83, 0.86)	0.54 (0.52, 0.55)	1.83
2430	*Staphylococcus aureus* (STH)	0.93 (0.91, 0.96)	0.53 (0.51, 0.56)	2.00
1875	*Streptococcus agalactiae*	0.91 (0.87, 0.94)	0.53 (0.50, 0.56)	1.93
2212	*Streptococcus uberis*	0.83 (0.81, 0.85)	0.53 (0.51, 0.55)	1.77
964	*Streptococcus dysgalactiae*	0.96 (0.93, 0.99)	0.53 (0.49, 0.56)	2.02
288	Major Gram-negative pathogens^b^	0.93 (0.90, 0.96)	0.52 (0.46, 0.58)	1.95
**11 426**	**Major pathogens**	**0.88 (0.87, 0.90)**	**0.58 (0.56, 0.59)**	**2.09**
445	*Staphylococcus pseudintermedius*	0.53 (0.48, 0.58)	0.52 (0.48, 0.57)	1.11
17 249	Coagulase-negative staphylococci	0.59 (0.59, 0.60)	0.55 (0.54, 0.56)	1.32
651	*Enterococcus faecalis*	0.85 (0.83, 0.88)	0.52 (0.49, 0.56)	1.87
249	*Other minor pathogens*^c^	0.80 (0.74, 0.85)	0.52 (0.46, 0.59)	1.66
379	*Other Gram-negative bacteria*^d^	0.77 (0.73, 0.82)	0.52 (0.47, 0.58)	1.62
**18 973**	**Minor pathogens**	**0.61 (0.60, 0.62)**	**0.56 (0.55, 0.57)**	**1.37**
**59 236**	**Culture-negative samples**	**0.36 (0.35, 0.38)**	**0.29 (0.28, 0.31)**	**0.52**

*Source*: Milk laboratory, Faculty of Veterinary Science, University of Pretoria

a , Confidence intervals have been adapted for clustering; LCI/UCI lower and upper confidence intervals;

b , major Gram-negative pathogens: *Escherichia coli, Serratia* spp. and *Klebsiella pneumonia*;

c , other minor pathogens: *Micrococcus* spp., *Enterococcus canis, Streptococcus pyogenes* and *Trueperella pyogenes*;

d , other Gram-negative bacteria: all Gram-negative bacteria isolated excluding *Escherichia coli, Serratia* spp. and *Klebsiella pneumonia.*

### Composite milk samples

Major Gram-positive, Gram-negative and minor pathogens were, respectively, isolated from 8.9%, 0.3% and 34.2% of composite samples examined. The most frequently isolated bacterial species in this sample type were CNS, *S. aureus* (STA and STH) and *S. uberis.* Of the samples examined, 40.1% of bacteria-positive samples and 62.3% of all examined had an SCC in excess of 200 000 cells/mL. Approximately, 80% of the major Gram-positive and Gram-negative groups and 51.7% of minor pathogen group had SCC exceeding 200 000 cells/mL. More than 80% of all major pathogens investigated in this study had SCC exceeding 200 000 cells/mL except *S. aureus* (STA) and *S. uberis*. In 81% of samples positive for *Trueperella pyogenes,* a minor pathogen, the SCC exceeded 200 000 cells/mL (Table 4).

**TABLE 4:** Composite milk samples either negative or positive for bacteria indicating individual pathogen-specific bacteria and groups isolated from samples with and without somatic cell counts above 200 000 cells/mL milk.

Bacteria isolated	Total numbers of samples where bacteria were isolated	As a % of the total samples	As a % of bacteria isolated	Number of samples with a SCC > 200 000 cells/mL	As a % of all cows with SCC > 200 000 cells/mL per bacterial species†	As a % of bacteriological diagnoses with SCC > 200 000 cells/mL
*Staphylococcus aureus* (STA)	9550	2.8	6.4	7188	5.2	75.3
*Staphylococcus aureus* (STH)	3972	1.2	2.7	3417	2.5	86.0
*Streptococcus agalactiae*	4759	1.4	3.2	4089	3.0	85.9
*Streptococcus uberis*	9173	2.7	6.1	7035	5.1	76.7
*Streptococcus dysgalactiae*	3159	0.9	2.1	2744	2.0	86.9
**Major Gram-positive pathogens**	**30 613**	**8.9**	**20.5**	**24 473**	**17.7**	**79.9**
*Escherichia coli*	341	0.1	0.2	325	0.2	95.3
*Klebsiella pneumonia*	282	0.1	0.2	271	0.2	96.1
*Serratia* spp.	240	0.1	0.2	233	0.2	97.1
**Major Gram-negative pathogens**	**863**	**0.3**	**0.6**	**829**	**0.6**	**96.1**
**Major pathogens**	**31 476**	**9.1**	**21.1**	**25 302**	**18.3**	**80.4**
*Staphylococcus pseudintermedius*	1458	0.4	1.0	685	0.5	47.0
*Coagulase-negative staphylococci*	111 461	32.3	74.6	56 706	40.9	50.9
*Streptococcus pyogenes*	127	0.0	0.1	97	0.1	76.4
*Enterococcus faecalis*	2603	0.8	1.7	1986	1.4	76.3
*Enterococcus canis*	159	0.1	0.1	103	0.1	64.8
*Trueperella pyogenes*	147	0.0	0.1	120	0.1	81.6
*Micrococcus* spp.	1006	0.3	0.7	508	0.4	50.5
*Other Gram-negative bacteria*^a^	986	0.3	0.7	824	0.6	83.6
**Minor pathogens**	**117 947**	**34.2**	**78.9**	**61.3**	**44.1**	**51.7**
All culture-negative samples	195 644	56.7	-	52 166	37.7	26.7
**All culture-positive samples**	**149 423**	**43.3**	-	**86 331**	**62.3**	**57. 8**
**All samples examined**	**345 067**	-	-	**138 497**	-	**40.1**

*Source*: Milk laboratory, Faculty of Veterinary Science, University of Pretoria

† , The number of composite milk samples, per pathogen group or species, that had somatic cell counts below 200 000 cells/mL expressed as a percentage of the total composite samples (138 497) identified with somatic cell counts below 200 000 cells/mL.

SCC, somatic cell counts.

In composite milk samples, 9.5% *S. dysgalactiae*, 10.9% *S. agalactiae,* 10.9% *S. aureus* (STA), 19.9% *S. aureus* (STH) and 40.6% CNS had SCC less than or equal to 150 000 cells/mL milk (Table 5).

**TABLE 5:** Cumulative percentages of 10 bacteria species and culture-negative samples isolated from composite cow milk samples distributed on various cell count thresholds.

SCC × 10^3^ cells/mL	% CNS	% STA	% STH	% STI	% SAG	% SUB	% SDY	% SFA	% SCA	% SPY	% No growth
50	15.3	7.3	3.5	17.4	3.1	6.7	2.4	6.8	12.0	6.3	39.5
100	29.6	14.3	7.4	32.3	6.6	12.4	5.9	12.7	17.0	12.6	56.9
150	40.6	19.9	10.9	44.3	10.9	18.3	9.5	18.4	26.4	18.9	66.8
200	49.1	24.7	14.0	53.0	14.1	23.3	13.1	23.7	35.2	23.6	73.3
250	55.9	28.8	16.7	58.9	17.2	28.3	16.4	28.3	37.1	26.8	77.5
300	61.4	32.3	18.8	64.4	20.2	32.5	19.1	33.2	39.6	33.1	80.6
350	65.7	35.3	21.1	68.3	23.5	36.3	22.1	37.9	43.4	40.2	82.9
400	69.3	37.8	23.0	71.8	26.1	39.9	25.2	41.8	48.4	42.5	84.7
450	72.2	40.1	25.1	74.4	28.2	43.3	27.9	45.1	49.7	44.9	86.3
500	74.8	42.2	26.9	76.2	30.3	46.0	31.3	48.4	52.2	48.0	87.6
750	82.7	50.5	33.5	82.3	39.0	57.3	42.9	60.4	61.6	62.2	91.4
Sample size	111 461	9550	3972	1458	4759	9173	3159	2603	159	127	195 644

*Source*: Milk laboratory, Faculty of Veterinary Science, University of Pretoria

SCC, somatic cell counts; CNS, coagulase-negative staphylococci; STA and STH, *Staphylococcus aureus*; STI, *Staphylococcus pseudintermedius*; SAG, *Streptococcus agalactiae*; SUB, *Streptococcus uberis*; SDY, *Streptococcus dysgalactiae*; SFA, *Enterococcus faecalis*; SCA, *Enterococcus canis*; SPY, *Streptococcus pyogenes*; no growth, culture-negative samples.

*n* = 345 461.

At a 200 000 cells/mL SCC threshold, the sensitivity for detecting major Gram-positive, Gram-negative and minor pathogens was 79.9%, 95.5% and 51.7% with specificity of 50.3%, 52.8% and 53.5%, respectively. At a 150 000 cells/mL SCC threshold, the sensitivity improved to 84.2%, 96.1% and 60.1% for the same microbial groups (Table 6).

**TABLE 6:** Sensitivity and Specificity with 95% lower and upper for various bacterial species and groups indicated in composite samples at somatic cell counts threshold levels of 150 000 cells/mL and 200 000 cells/mL.

Bacteria isolated	Sensitivity at 150 000 cells/mL threshold (95% LCI and UCI)	Specificity at 150 000 cells/mL threshold (95% LCI and UCI)	Sensitivity at 200 000 cells/mL threshold (95% LCI and UCI)	Specificity at 200 000 cells/mL threshold (95% LCI and UCI)
*Staphylococcus aureus* (STA)	0.80 (0.78, 0.82)	0.52 (0.51, 0.53)	0.74 (0.73, 0.77)	0.52 (0.52, 0.53)
*Staphylococcus aureus* (STH)	0.89 (0.86, 0.92)	0.53 (0.52, 0.53)	0.86 (0.83, 0.89)	0.525 (0.52, 0.53)
*Streptococcus agalactiae*	0.89 (0.87, 0.92)	0.52 (0.52, 0.53)	0.86 (0.83, 0.89)	0.525 (0.52, 0.53)
*Streptococcus uberis*	0.82 (0.80, 0.84)	0.52 (0.51, 0.53)	0.77 (0.75, 0.79)	0.523 (0.52, 0.53)
*Streptococcus dysgalactiae*	0.91 (0.87, 0.94)	0.53 (0.52, 0.53)	0.87 (0.84, 0.90)	0.53 (0.52, 0.54)
**Major Gram-positive Pathogens**	**0.84 (0.81, 0.88)**	**0.50 (0.49, 0.51)**	**0.78 (0.77, 0.83)**	**0.50 (0.50, 0.51)**
**Major Gram-negative pathogens^a^**	**0.96 (0.90, 1.03)**	**0.53 (0.52, 0.54)**	**0.96 (0.89, 1.02)**	**0.53 (0.52. 0.54)**
*Staphylococcus pseudintermedius*	0.56 (0.52, 0.60)	0.53 (0.52, 0.54)	0.47 (0.44, 0.51)	0.53 (0.52, 0.54)
*Coagulase-negative staphylococci*	0.59 (0.58, 0.61)	0.50 (0.49, 0.51)	0.51 (0.50, 0.52)	0.54 (0.53, 0.55)
*Enterococcus faecalis*	0.82 (0.78, 0.85)	0.53 (0.52, 0.54)	0.76 (0.73, 0.80)	0.53 (0.52, 0.54)
Other Gram-negative bacteria^b^	0.83(0.77, 0.89)	0.53 (0.52, 0.54)	0.84 (0.78, 0.89)	0.53 (0.52, 0.54)
*Streptococcus pyogenes*	0.81 (0.67, 0.98)	0.53 (0.52, 0.54)	0.76 (0.62, 0,93)	0.53 (0.52, 0.54)
*Enterococcus canis*	0.74 (0.61, 0.88)	0.53 (0.52, 0.54)	0.65 (0.53, 0.78)	0.53 (0.52, 0.54)
*Trueperella pyogenes*	0.87 (0.73, 1,03)	0.53 (0.52, 0.54)	0.82 (0.68, 0.97)	0.53 (0.52, 0.54)
*Micrococcus* spp.	0.59 (0.54, 0.64)	0.53 (0.52, 0.54)	0.51 (0.46, 0.55)	0.53 (0.51, 0.54)
Other minor pathogens combined^c^	0.65 (0.61, 0.70)	0.53 (0.52, 0.54)	0.58 (0.54, 0.62)	0.53 (0.52, 0.54)
**Minor pathogens**	**0.60 (0.59, 0.62)**	**0.49 (0.48, 0.50)**	**0.52 (0.51, 0.53)**	**0.54 (0.53, 0.55)**
**All bacteria isolated**	**0.65 (0.64, 0.67)**	**0.67 (0.66, 0.68)**	**0.58 (0.57,0.59)**	**0.73 (0.72, 0.75)**

*Source*: Milk laboratory, Faculty of Veterinary Science, University of Pretoria

a , Major Gram-negative pathogens: *Escherichia coli, Klebsiella pneumonia* and S*erratia* spp.;

b , other Gram-negative bacteria: all Gram-negative isolated excluding *Escherichia coli, Klebsiella pneumonia* and *Serratia* spp.;

c , other minor pathogens combined: *Streptococcus pyogenes, Enterococcus canis, Trueperella pyogenes and Micrococcus* spp.

In almost all cases in composite samples, the probability of identifying pathogen groups or specific pathogen species was only slightly higher than the likelihood of identifying culture-negative samples at both 150 000 cells/mL and 200 000 cells/mL SCC thresholds. *Staphylococcus pseudintermedius* was less likely to be identified than culture-negative samples when using an SCC threshold of 200 000 cells/mL (Table 7).

**TABLE 7:** The positive likelihood ratios at somatic cell count thresholds of 150 000 cells/mL and 200 000 cells/mL for detecting various bacterial species and groups in composite samples.

Bacteria isolated	Positive likelihood ratio at 150 000 cells/mL	Positive likelihood ratio at 200 000 cells/mL
*Staphylococcus aureus* (STA)	1.67	1.54
*Staphylococcus aureus* (STH)	1.88	1.81
*Streptococcus agalactiae*	1.87	1.81
*Streptococcus uberis*	1.71	1.61
*Streptococcus dysgalactiae*	1.91	1.83
**Major Gram-positive pathogens**	**1.68**	**1.61**
**Major Gram-negative pathogens^a^**	**2.04**	**2.02**
*Staphylococcus pseudintermedius*	1.18	1.00
Coagulase-negative staphylococci	1.18	1.10
*Enterococcus faecalis*	1.73	1.61
Other Gram-negative bacteria^b^	1.75	1.77
**Minor pathogens**	**1.18**	**1.11**
**Bacteria pooled**	**1.97**	**2.17**

*Source*: Milk laboratory, Faculty of Veterinary Science, University of Pretoria

a , Major Gram-negative pathogens: *Escherichia coli, Klebsiella pneumonia* and S*erratia* spp.;

b , other Gram-negative bacteria: all Gram-negative isolated excluding *Escherichia coli, Klebsiella pneumonia* and *Serratia* spp.

## Ethical considerations

There were no animals used in these trials; only milk samples were taken for routine analysis. An application was submitted and approval was obtained from the Animal Ethics Committee (AEC) of the University of Pretoria.

## Discussion

This study provided an overview of the incidence of IMIs and culture-positive samples under operational conditions experienced by South African commercial dairy herds at the time of the investigation. This study was intended as an initial investigation on data already available from routine samples.

### Quarter milk samples

Observations of quarter milk are not independent and within-cows dependency was considered in the study design. The within-herd correlations were not taken into account in this initial study to enable evaluation of results as they are obtained under field conditions on a daily bases by diagnostic laboratories. The ratio of culture-negative to culture-positive quarter milk samples with SCC less than or equal to 200 000 cells/mL was approximately four to one, whereas almost equal numbers of culture-negative and culture-positive samples were present at SCC exceeding 200 000 cells/mL (Table 1). Although the ratio of positive cultures increased with increasing SCC conforming to general accepted standards (Biggs 2009; National Mastitis Council 2001; Roy et al. 2012), it was surprising to learn that such a large portion of samples with SCC exceeding 200 000 cells/mL were culture negative (Table 1). This finding brings into question the use of the SCC test as a survey tool to identify IMI under field conditions (Table 1). Although numerous factors can influence the SCC at individual cow- and udder-quarter level, such as parity, lactation stage, incorrect milking machine settings, stress and other factors including genetics, the most important cause remains the infection status of the mammary gland (Schepers et al. 1997).

A further objective of this study was to determine whether the SCC levels differed in milk samples identified with different pathogen species and between pathogen groups. Although only very small percentages of major Gram-negative bacteria (*Escherichia coli, Klebsiella pneumonia* and *Serratia* spp.) were isolated from South African dairy herds, most were associated with high SCC (Table 1). In contrast to the finding of Harmon (1994) that quarters with SCC below 200 000 cells/mL were not likely to be infected with major mastitis pathogens, we isolated 11.0% of major pathogens from low SCC samples (Table 1). This value could even be slightly higher as samples that yielded a mixed growth of bacteria were not included in this study. Few researchers in other countries sample as a rule all lactating cows in herds for microbiological evaluation, but rather rely on identification of clinical mastitis and cases with high SCC to identify problem animals. Cows that are positive for major pathogens with low SCC in the milk samples may therefore pass unnoticed. In herds with poor parlour hygiene and management, the risk of transmission from unidentified cows causing new IMI can be high (Sears et al. 1990; Zadoks et al. 2002).

Malinowski et al. (2006) found very high SCC (some exceeding 10 million cells/mL) for the Gram-negative pathogen group, *S. agalactiae* and *T. pyogenes*, whereas Schepers et al. (1997) identified *S. aureus* as being responsible for the highest SCC. In this study from the eight pathogens that were quantified according to SCC levels, *S. dysgalactiae* had overall the highest SCC followed by *S. aureus* (STH and STA) and *S. agalactiae* (Table 2). Although only small numbers of *T. pyogenes* were isolated in this data set, more than 84% of these bacteria had SCC exceeding 200 000 cells/mL (Table 1). Another bacterium, *Streptococcus pyogenes*, which is not often isolated from cow’s milk but is frequently isolated from human throat swabs and is known to cause reverse zoonosis (Aboul Dahab, Darhous & Abdel Rahman 1993), was found to be responsible for high SCC (Table 1).

A study conducted by Nickerson and Boddie (1994) showed that the mean SCC for samples identified with the CNS strains *Staphylococcus chromogenes* and *Staphylococcus hyicus* was 168 000 cells/mL and 193 000 cells/mL compared with 39 000 cells/mL from uninfected quarters. In this study, CNS were the most frequently isolated bacteria and were equal in numbers to all the major pathogens combined. More than 61% of samples with minor pathogens had SCC greater than or equal to 200 000 cells/mL (Table 1). This finding indicates the extent to which minor pathogens may contribute towards high bulk milk cell counts. Malinowski et al. (2006) indicated that most milk samples with CNS, *S. aureus* and streptococcal IMI had SCC ranging from 200 000 cells/mL to 2 million cells/mL. Of the more than 17 000 milk samples from which CNS was isolated in this study, 60% had SCC above 200 000 cells/mL, and of those, 26.7% had SCC exceeding 750 000 cell/mL (Tables 1 and 2). Currently, CNS is still generally regarded as a minor udder pathogen, but scientists may need to reconsider this status in future when more information regarding pathogenicity of different CNS stains become available.

The sensitivity for detecting pooled udder pathogens at a 200 000 cells/mL SCC threshold was low (70.08%), but it was moderately high (88.2%) for detecting major udder pathogens (Table 3). Sears et al. (1990) found a 74.5% sensitivity at 200 000 cells/mL for a single bacterial culture to detect *S. aureus*. This sensitivity improved to 94% and 98% when two or three consecutive samples of the same quarter were evaluated. Under field conditions, cows are usually selected for culling based on the evaluation of single samples but follow-up samples should preferably be examined to improve diagnostic accuracy. The sensitivity of SCC for different minor pathogens varies from as low as 52.8% for *S. pseudintermedius* to 85.4% in the case of *Enterococcus faecalis* (Table 3).

The specificity at a 200 000 cells/mL SCC threshold to indicate the absence of major (57.7%) and minor (55.6%) pathogen groups was low. These values were not in agreement with results obtained by Dinsmore et al. (1990) of 95% – 100% for *S. agalactiae.* Although sample dependency of quarter SCC values within each cow was modelled, cow factors did not affect the outcome of results.

Positive likelihood ratios were used to indicate the probability of a sample being found accurately positive for specific bacteria when the SCC threshold would be increased when samples were accurately positive for specific bacteria. It was disappointing that at the 200 000 cells/mL SCC threshold, only the major pathogen group and *S. dysgalactiae* had a small increase in probability of detection and this applied to none of the minor pathogens. Even when all bacteria were pooled, the probability remained small at just above 2% (Table 3).

### Composite milk samples

The use of quarter milk samples for routine udder health monitoring has become expensive for large dairy herds, and initial udder health surveys are now conducted using composite cow milk samples in South African dairy herds (Petzer et al. 2017). It is important, therefore, to know how reliable SCC of composite milk samples are as indicators of IMI and culture-positive results and to learn more regarding the interpretation of these results. Although a composite milk sample is a combination of four quarters, a number of studies have found their SCC to be a useful indicator in mastitis control practices (Barkema et al. 1998, 1999; Bartlett et al. 1992; Erskine et al. 1987; Reyher & Dohoo 2011).

In both quarter and composite samples with SCC less than or equal to 200 000 cells/mL, the ratio for culture-negative to culture-positive samples was 7:3. In samples with SCC exceeding 200 000 cells/mL, composite samples had a ratio of one culture-negative sample per two culture-positive samples compared with a 1:1 ratio found in quarter milk samples (Tables 1 and 4). In contrast to results for quarter milk samples, a minority of both infected (44.4%) and uninfected (26.7%) composite samples had SCC exceeding 200 000 cells/mL. This indicated that this threshold might not be ideal to use in surveying composite milk samples for culture-positive samples. (Table 4). In order to use SCC in composite milk samples as an indicator of pathogen-specific diagnosis, an SCC threshold with results that compare more favourably with the 200 000 cells/mL of quarter milk samples is needed.

Most samples with *S. aureus* (STA and STH), *S. agalactiae, S. uberis* and *S. dysgalactiae* had SCC exceeding 200 000 cells/mL in both sample types but the levels at which they were isolated were noticeably lower in composite samples (Tables 1 and 4). *Staphylococcus aureus* has been shown in the past to cause a high incidence of mastitis and to be an important cause of continuing udder infections (Barkema, Schukken & Zadoks 2006). The highest SCC was evident in this study in culture-positive samples containing *S. aureus* (STH), *S. dysgalactiae* and *S. agalactiae* (Table 5). Mastitis and IMI caused by CNS have increased in recent years, and SCC in CNS-positive samples have risen significantly (Reksen, Sølverød & Østerås 2008; Reyher et al. 2012). *Staphylococcus pseudintermedius* appears to be an emerging udder pathogen in South African dairies but was present in samples with low SCC (≤ 200 000 cells/m) both in quarter (46.7%) and composite samples (53.0%) (Tables 2 and 5).

Petzer et al. (2017) have found that an SCC threshold of 150 000 cells/mL was more appropriate to use in composite milk samples when surveying a herd for the purpose of identifying samples with positive bacterial growth. This study showed that the percentages of different pathogen species that were isolated from composite milk samples at a 150 000 cells/mL SCC threshold compared better than the percentages isolated at a 200 000 cells/mL threshold to that isolated from quarter milk samples at an SCC threshold of 200 000 cells/mL. When lowering the threshold to 150 000 cells/mL, an additional 8% of CNS, *S. pseudintermedius* and *Enterococcus canis* could be identified and more than 4% extra infections of *S. uberis, S. aureus* (STA and STH) and *S. agalactiae* (Table 5). The bacteria isolated from composite milk samples with the highest SCC were *S. aureus* (STH), *Str. agalactiae, Str. dysgalactiae* and *Str. agalactiae* (Table 5). When only samples with an SCC exceeding 150 000 cells/mL are cultured, 31% of *S. aureus* (STA and STH), 18% of *Str. uberis*, 11% *S. agalactiae* and 10% of *S. dysgalactiae* would remain unidentified (Table 5). All of these bacteria are known to be contagious, and culture-negative cows milked after them may be at risk of being infected (Petzer et al. 2017). Decision-makers in the field may set their goal to eradicate *S. agalactiae* from a herd and, therefore, culture samples from all cows. Missing even one cow may start new infections or outbreaks in herds (Petzer et al. 2017). In the case of *S. uberis,* eradication is not an option as this is an environmental udder pathogen and only those with high SCC that are repeatedly isolated from the same cow would be important to identify to achieve the goal set here.

Although Reksen et al. (2008) found sensitivity in composite samples to be low, this study found moderately high to high sensitivities (84.2% and 96.1%) for identifying major Gram-positive and Gram-negative pathogens at the 150 000 cells/mL threshold but low sensitivity (65.3%) for detecting the minor pathogen group (Table 6). Lam et al. (1996) and Reyher and Dohoo (2010) reported at the 200 000 cells/mL threshold sensitivities of 63.0% – 77.1% to detect *S. aureus.* To detect *S. aureus* (STA) and (STH), we recorded sensitivity levels of 73.5% and 86.0%, respectively, at 200 000 cells/mL SCC, and these improved to 80.1% and 89.1% at 150 000 cells/mL SCC, respectively (Table 6). The sensitivity at 150 000 cells/mL for detecting *S*. *dysgalactiae* and *S. uberis* was 90.5% and 81.7% compared with 73.4% and 62.1% reported by Reyher and Dohoo (2010) at a 200 000 cells/mL SCC threshold. These findings supported the use of a 150 000 cells/mL SCC threshold in composite milk samples for detecting the udders infected with major pathogens.

There was little improvement in the level of specificity of the SCC test when using the lower threshold (Table 6). According to Reyher and Dohoo (2010), factors such as parity and days in milk did not influence sensitivity and specificity of composite sampling, only for CNS.

The positive likelihood ratio for detecting specific pathogens species and groups differed very little when the SCC was lowered from 200 000 cells/mL to 150 000 cells/mL milk (Table 7).

## Conclusion

Electronic somatic cell counting has traditionally been used since the 1960s as a diagnostic tool in udder health assessment (Heeschen 2010). Although most major and minor udder pathogen groups were isolated at SCC exceeding 200 000 cells/mL, over a third in quarter samples and almost a third in composite samples were culture negative at SCC exceeding this level. From these results, it is clear that SCC cannot be used on its own as a diagnostic tool to identify bacteria-positive cultures in quarter or composite samples. When opting to use SCC as a test indicating specific udder pathogens or groups, the low specificity of the test that was recorded will have a negative effect on the accuracy of results. However, decreasing the SCC threshold in composite samples to 150 000 cells/mL improved the sensitivity of the test to moderately high and high levels of 84% and 96% for the major Gram-positive and Gram-negative udder pathogen groups, whereas specificity remained low at 50% and 52%, respectively.

The level of SCC differed considerably according to the various udder pathogens identified, and species that caused the highest SCC in both sample types were *S. dysgalactiae, S. agalactiae* and *S. uberis.* When SCC thresholds of 200 000 (in quarter milk samples) and 150 000 cells/mL (in composite milk samples) were used to detect *S. aureus* positive cultures, it was found that 20.5% of quarters and 30.8% of cows positive for *S. aureus* might remain undetected due to their low SCC. These values could increase to 49% quarters and 69% of cows if milk samples with SCC exceeding 500 000 cells/mL were to be cultured. This article contributes to knowledge of the estimated risk of under-diagnosing of pooled bacterial infections, various pathogens and pathogen groups at particular SCC levels in both quarter and composite milk samples. The knowledge gained in this study can lead to efficient more goal-orientated decision-making.

The effects of days in lactation, parity, milk yield, management level and prevalence of pathogen-specific IMI or culture-positive samples on SCC thresholds were not measured in this research and need to be further investigated.
